# Optical Coherence Tomography as a Tool for Ocular Dynamics Estimation

**DOI:** 10.1155/2015/293693

**Published:** 2015-10-18

**Authors:** Damian Siedlecki, Waldemar Kowalik, Henryk Kasprzak

**Affiliations:** Department of Optics and Photonics, Wroclaw University of Technology, Wybrzeze Wyspianskiego 27, 50-370 Wroclaw, Poland

## Abstract

*Purpose*. The aim of the study is to demonstrate that the ocular dynamics of the anterior chamber of the eye can be estimated quantitatively by means of optical coherence tomography (OCT). *Methods*. A commercial high speed, high resolution optical coherence tomographer was used. The sequences of tomographic images of the iridocorneal angle of three subjects were captured and each image from the sequence was processed in MATLAB environment in order to detect and identify the contours of the cornea and iris. The data on pulsatile displacements of the cornea and iris and the changes of the depth of the gap between them were retrieved from the sequences. Finally, the spectral analysis of the changes of these parameters was performed. *Results*. The results of the temporal and spectral analysis manifest the ocular microfluctuation that might be associated with breathing (manifested by 0.25 Hz peak in the power spectra), heart rate (1–1.5 Hz peak), and ocular hemodynamics (3.75–4.5 Hz peak). *Conclusions*. This paper shows that the optical coherence tomography can be used as a tool for noninvasive estimation of the ocular dynamics of the anterior segment of the eye, but its usability in diagnostics of the ocular hemodynamics needs further investigations.

## 1. Introduction

For over 20 years, optical coherence tomography has evolved from an emerging imaging technology of uncertain future to a well-established diagnostic tool being used in many different fields of medicine. Due to its unique features of visualizing transparent and semitransparent objects with an ultrahigh resolution, it has found the greatest interest in ophthalmology, particularly in imaging of the fine structures of the cornea [1, 2] and the retina [3–5]. Furthermore, a significant development of the instrumentation and data processing techniques [6–8] has opened OCT to brand new applications in ocular imaging, measurement, and diagnostics, hardly achievable with other technologies.

Ocular dynamics is a complex phenomenon which is related to the hemodynamics (pulse) [9–12] and mechanical parameters of the eye globe [13–20]. It has been shown before that the rapid variations of the intraocular pressure (IOP), known as ocular pulse, are manifested by the axial and radial displacements of the corneal surface [9] as the most external and the easiest to access structure of the eye globe. Also the simultaneous micromovements of the retina were observed as a result of the ocular pulsation [21–23]. Microfluctuation of the iris and/or crystalline lens that might be affected by the intraocular pressure variation related to the retinal blood flow was reported in other studies, as well [24].

In the existing literature, the term “dynamics” used in relation to the iridocorneal angle is usually associated either with its changes with light-dark adaptation [25, 26] or drug application [27], and in both of these cases the tomographic images of the angles are captured as a single shot before and after changing the conditions of the experiment. The aim of the current study is to present a new approach to the dynamic changes of the anterior segment of the eye. In this approach it can be continuously examined in a short period of time, with a relatively high frequency (of more than 20 Hz). The short-term microfluctuation of the anterior chamber angle can be successfully visualized by means of a high speed spectral domain OCT (SD-OCT). The current paper presents also a preliminary example of the analysis of these pulsatile fluctuations, including their spectral analysis, related to the breathing and blood pulsation.

## 2. Methods

### 2.1. In Vivo Measurements

Three patients, aged from 65 to 70, participated in the study. Subject #1 was diagnosed with angle closure glaucoma (ACG) and Subjects #2 and #3 were diagnosed with open-angle glaucoma (OAG). The iridocorneal angle in the temporal quadrant of their eyes (OS for Subject #1, OD for Subjects #2 and #3) was imaged with use of a SD-OCT Copernicus HR (Optopol Technology, Zawiercie, Poland). It is a high axial resolution (up to 3 *μ*m in tissue), high speed (up to 52 000 A-scans/sec, according to the device specifications) spectral domain OCT, using a 850 nm superluminescent diode (SLED) as a light source. It is optionally equipped with an attachable anterior segment adapter, which makes it useful for the anterior chamber measurement purposes. The standard examination mode denoted as “anterior” and the scanning protocol denoted as “animation” were used. This particular protocol enables capturing the sequence of cross-sectional images (B-scans) in the same meridional position. The length of time of the sequence depends on the lateral density of a single image (the number of A-scans contributed to a single B-scan). The maximum number of A-scans in this scanning protocol is 1800. This enables the capturing of the sequence of 3.96 s long (as estimated by the standard software distributed together with the device), containing a series of 90 images of the iridocorneal angle, in the same meridional cross-section. This configuration was used for the purposes of this study.

During the measurements the subject was fixating centrally on the internal target and his head was stabilized in the chin- and head-rest of the device. He was allowed to breathe freely and was asked not to blink during the sequence acquisition.

All patients provided written informed consent before enrolling in the study. The measurement protocol had been approved by the Institutional Review Board and met the tenets of the Declaration of Helsinki.

### 2.2. Data Processing

**Figure 1:**
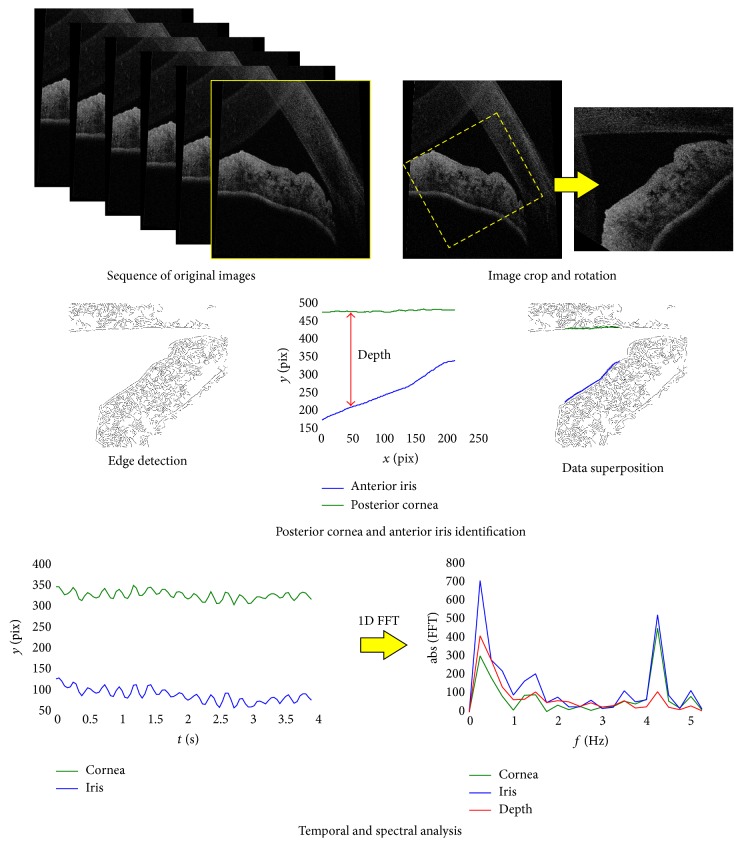
The stages of the image processing for the reconstruction of the posterior surface of the cornea and the anterior surface of the iris. The effect of the “bended” cornea on the original image is an artefact that can be associated with the Fourier transform of the optical signal reaching the light detector and the relatively short axial range of the instrument (c.a. 2.5 mm). The image of superposition of the identified surfaces and data after edge detection is only for demonstration purposes.

The results of the measurement for each patient contained a series of 90 consecutive cross-sectional images of a resolution 900 × 1009 pixels, captured with the frequency of 22.7 Hz (90 frames captured within 3.96 s). Each image was processed in MATLAB with use of a custom-developed script. The semiautomatic procedures included (a) the selection of the area of interest and its rotation so that the cornea was situated in the upper part of the image; (b) the edge detection [28]; and (c) the identification of the posterior surface of the cornea and the anterior surface of the iris. Because of the different scattering properties of the cornea and iris (the cornea has lower signal-to-noise ratio than the iris), several existing algorithms [29–31] of edge and contour detection needed to be tested with various parameters, before the results presented in the next section were obtained. The horizontal range, in which the surfaces of the cornea and iris could be unerringly identified automatically, was selected subjectively by the software operator. The coordinates of the start and end points of this range depended on the observed movability of the ocular structures in the whole sequence. The stages of the image processing used in the study are presented in Figure 1.

After identification of the posterior cornea and anterior iris surfaces on each particular scan from the sequence, it was possible to show variability of their temporal position. Their observed displacements might be associated with the fast, dynamic changes of either the internal conditions of the eye globe, such as ocular pulse and muscle contraction/relaxation, or external ones (i.e., breathing). For this purpose, the depth *D* of the gap between the posterior surface of the cornea and anterior surface of the iris along each pixel column in the rotated image was calculated in terms of pixels. The spectral analysis of the variability of the position in time was performed as well, as one-dimensional fast Fourier transform (FFT) of the temporal changes of the location of the identified structures (cornea and iris and its longitudinal separation) in each particular pixel column. As a result, the spectral map of the fluctuations in position of each pixel was plotted.

## 3. Results

The sequences showing the dynamic fluctuations of the iridocorneal region captured for all the subjects participating in the study can be seen on the videos (see Video, Supplemental Digital Content 1, captured for Subject #1; see Video, Supplemental Digital Content 2, captured for Subject #2; see Video, Supplemental Digital Content 3, captured for Subject #3, in Supplementary Material available online at http://dx.doi.org/10.1155/2015/293693). The frame per second (fps) rate of the videos is two times lower than the acquisition rate of the OCT instrument.

**Figure 2:**
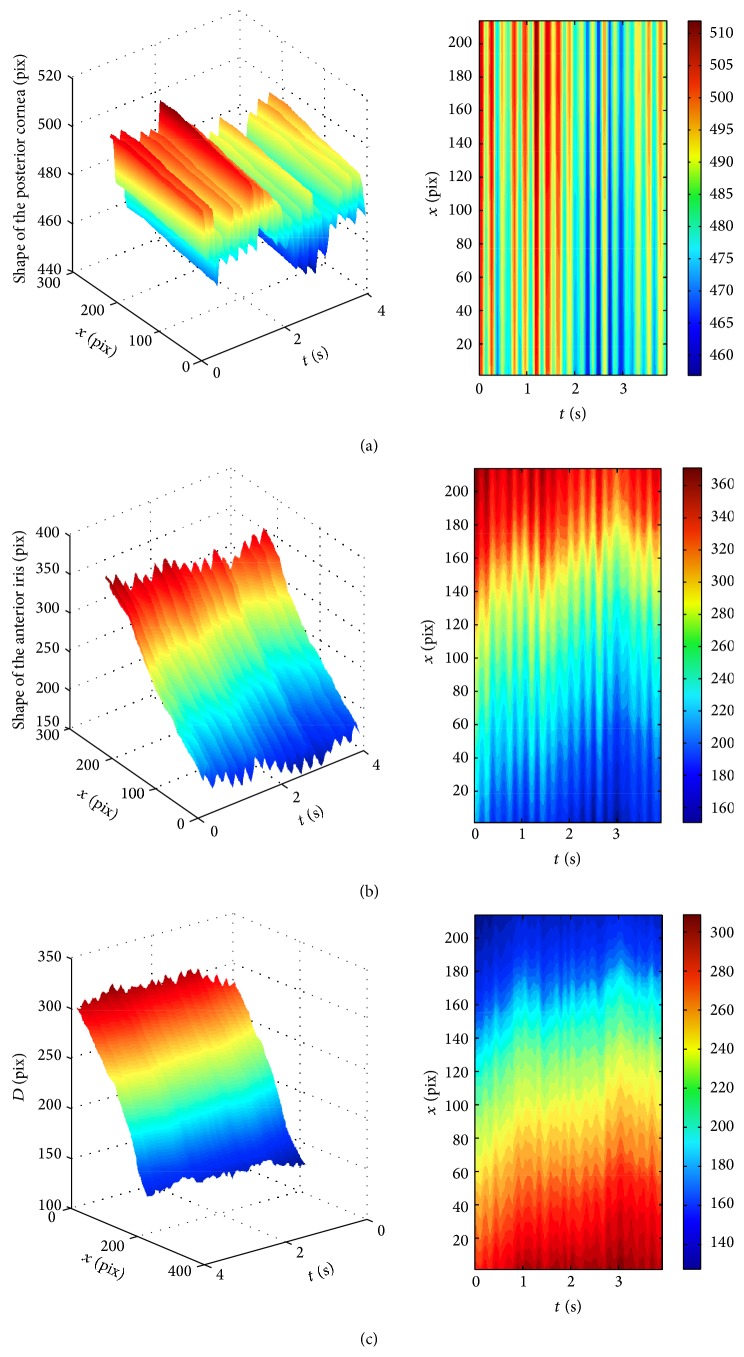
An example of temporal fluctuation of the (a) posterior cornea, (b) anterior iris, and (c) depth *D* of the gap between the cornea and the iris being a result of the data processing of the sequence captured for Subject #1. Both plots in the row represent the same data, but the pulsations are better demonstrated in the contour plots.

**Figure 3:**
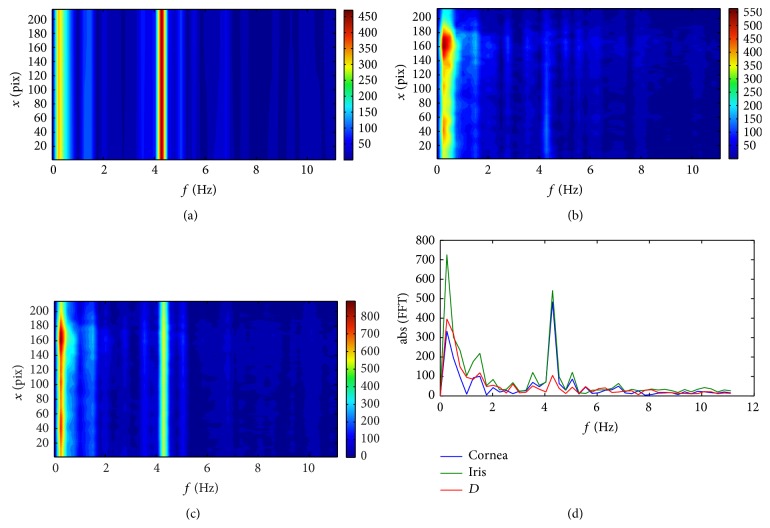
Fourier spectra of displacement data obtained from the sequence captured for Subject #1: (a) the results for the posterior surface of the cornea; (b) the results for the anterior surface of the iris; (c) the results for the distance *D* between the cornea and iris; (d) the power spectra averaged over the *x* coordinate. The spectral analysis was performed as the one-dimensional fast Fourier transform of the temporal changes of the surfaces.

**Figure 4:**
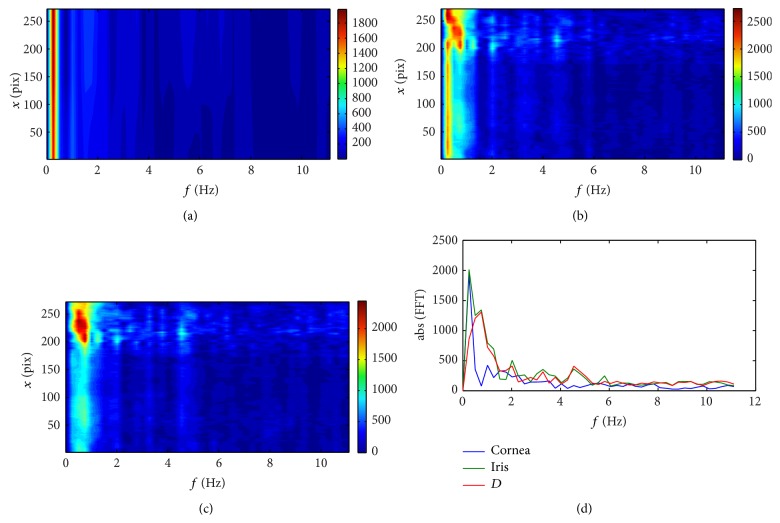
Fourier spectra of displacement data obtained from the sequence captured for Subject #2: (a) the results for the posterior surface of the cornea; (b) the results for the anterior surface of the iris; (c) the results for the distance *D* between the cornea and iris; (d) the power spectra averaged over the *x* coordinate. The spectral analysis was performed as the one-dimensional fast Fourier transform of the temporal changes of the surfaces.

**Figure 5:**
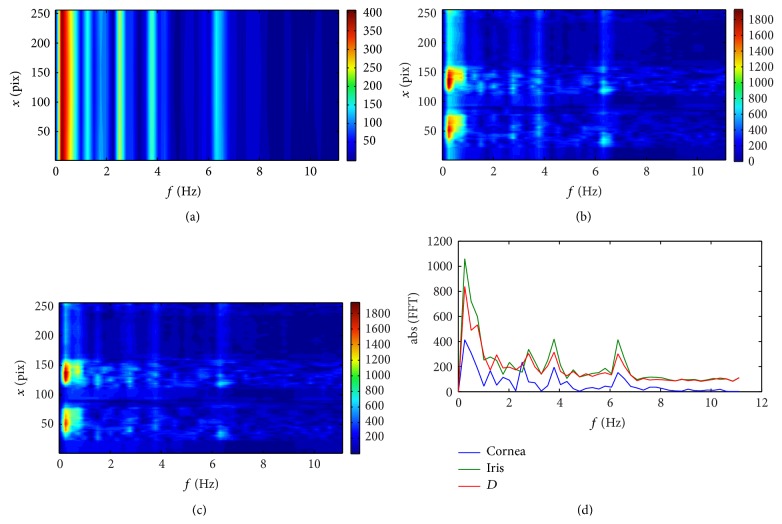
Fourier spectra of displacement data obtained from the sequence captured for Subject #3: (a) the results for the posterior surface of the cornea; (b) the results for the anterior surface of the iris; (c) the results for the distance *D* between the cornea and iris; (d) the power spectra averaged over the *x* coordinate. The spectral analysis was performed as the one-dimensional fast Fourier transform of the temporal changes of the surface.

Figure 2 presents the microfluctuation of the cornea and iris captured for Subject #1. At first glance, the same behavior can be observed for all the subjects participating in the study. The depth *D* between the posterior surface of the cornea and the anterior surface of the iris undergoes some small changes, as well, which indicates small difference between the amplitudes of the pulsatile movements of the cornea and the iris. However, the spectral analysis shows slightly different characteristics of these movements for each patient, which can be seen in Figures 3–5.

It needs to be emphasized that the results presented here are not the results of the measurements of the iridocorneal angle itself, which are expressed in terms of angles. The results presented above are rather the results of the measurements of the distance between the posterior cornea and anterior surface of the iris, expressed in pixels. Of course, they are associated with the iridocorneal angle; however, the tomographic images were not corrected from the optical distortion, and instead of using the units of angles and micrometers, it was more appropriate to use pixels.

## 4. Discussion

There are three basic features that are manifested in all the spectral plots for all the subjects. The first one is the peak for the low frequency (about 0.25 Hz) that is likely associated with breathing [32, 33] and respective head movement during measurement [34].

The second feature is the small peak that appears in the frequency range between 1 and 1.5 Hz. These frequencies can be linked with blood pulsation in the ocular vessels [32, 33]. An interesting fact is that for Subject #1 this peak is quite distinguishable for both the posterior cornea and anterior iris and the distance *D* between them. For Subject #3 this peak is visible for cornea and iris, but not for the depth *D*, whereas for Subject #2 this peak moves slightly from about 1 Hz for the cornea to about 0.75 Hz for the iris and the distance *D*, and for the *D* its magnitude is even higher than the peak associated with breathing. It can be the effect of the extensive peak of a large amplitude, which appears for pixel numbers larger than 200. This is the region of a step-like structure on the surface of the iris. Its lateral movements are manifested as changes of a very large amplitude in depth. This peak seems to cover (overlap) the neighboring frequencies which results in a frequency drift in the averaged spectrum for iris and depth noticeable in Figure 4(a).

The third common feature is the local, well distinct peak, with the maximum for the frequencies varying from 3.75 Hz (for Subject #3) to 4.5 Hz (for Subject #2). These peaks can be possibly related to the harmonics of the blood pulsation. For example, the results for Subject #3 (Figure 5) present the local peaks at frequencies 1.25, 2.5, 3.75, and 6.25 Hz, which are multiples of 1.25 Hz. For other subjects these harmonics are not so obvious, probably due to low sampling rate in the frequency domain or due to nonstationarity of the signal. In order to improve this analysis one should use OCT with possibility of recording longer sequences. As shown in the paper [34] the involuntary head movement can also influence the appearance of higher harmonics in the cornea position.

Some recent studies reported the phenomenon of ocular pulse dicrotism [35, 36], which is related to the condition (rigidity) of the arterial vessels and can be observed in older subjects. Since all the subjects in the current study are aged from 65 to 70, it is likely that the ocular pulse dicrotism could also be related to our observations. Further studies should give an answer, where explanation is more probable.

The number of the peaks that can be clearly and uniquely distinguished in the spectral characteristics of the movement of the anterior segment of the eye is different for various subjects. The origin of the maxima other than the one described above is not clear yet and can be the subject of another study.

## 5. Conclusions

This paper is a preliminary study of ocular dynamics, its imaging, and analysis. To the best of the authors' knowledge, this paper is the very first demonstration of the rapid dynamic fluctuations of the geometry of the iridocorneal part of the eye. The imaging was performed with use of a commercially available instrument with high acquisition rate.

The power spectra of the pulsatile micromovements of the whole anterior segment can provide some information about individual parameters of a subject eye. These parameters are the mechanical response of the ocular structures on the pulsation in the vitreous body and may undergo some discrete changes with the progress of the pathological states. However, the potential of the method as a tool in early diagnosis of the ocular diseases, in particular glaucoma, is still to be studied.

One of the problems that has to be overcome for the further development of the method is the stabilization of the images from the sequence of the tomograms, because some undesired lateral movements of the eye can be seen on the movies presented in videos. The significance of these lateral movements on the overall spectral characteristics was not the subject of the current study, and it was not estimated. However, this problem can be solved numerically, when the stabilization of each particular tomogram is performed. The development of a fully automatic image segmentation and surface identification [37–39] would also be helpful.

Further research on larger population samples with simultaneous use of other diagnostic techniques (i.e., pulse oximetry) would give an answer to the question of whether the presented method of ocular dynamics estimation by means of optical coherence tomography might be used for the diagnostics of ocular disorders associated with hemodynamics of the eye, such as glaucoma.

## Supplementary Material

The sequences showing the dynamic changes of the irido-corneal region captured for a) Subject #1, OS, temporal quadrant (multimedia file #1); b) Subject #2, OD, temporal quadrant (multimedia file #2); c) Subject #3, OD, temporal quadrant (multimedia file #3). The fps rate of the movies is two times lower than the acquisition rate of the OCT instrument.
